# Myelination sustains axonal bioenergetics by storing and consuming oxygen for aerobic metabolism

**DOI:** 10.1073/pnas.2526705123

**Published:** 2026-08-24

**Authors:** Isabella Panfoli, Giovanni Candiano, Maurizio Bruschi

**Affiliations:** ^a^ Department of Pharmacy, University of Genoa, Genoa 16132, Italy; ^b^ Unit of Nephrology, Dialysis and Transplantation, Laboratory of Molecular Nephrology, Istituto Giannina Gaslini, Scientific Institute for Research, Hospitalization and Healthcare, Genoa 16147, Italy; ^c^ Department of Experimental Medicine, University of Genoa, Genoa 16132, Italy

Vervust et al. beautifully propose that myelin sheaths may act as oxygen reservoirs, supporting mitochondrial function in axons (1). This thought-provoking model, grounded in molecular dynamics simulations, suggests that myelin, modeled as a network of resistor-capacitor circuits, provides a store of diffusible oxygen to sustain neuronal metabolism.

**Fig. 1.**
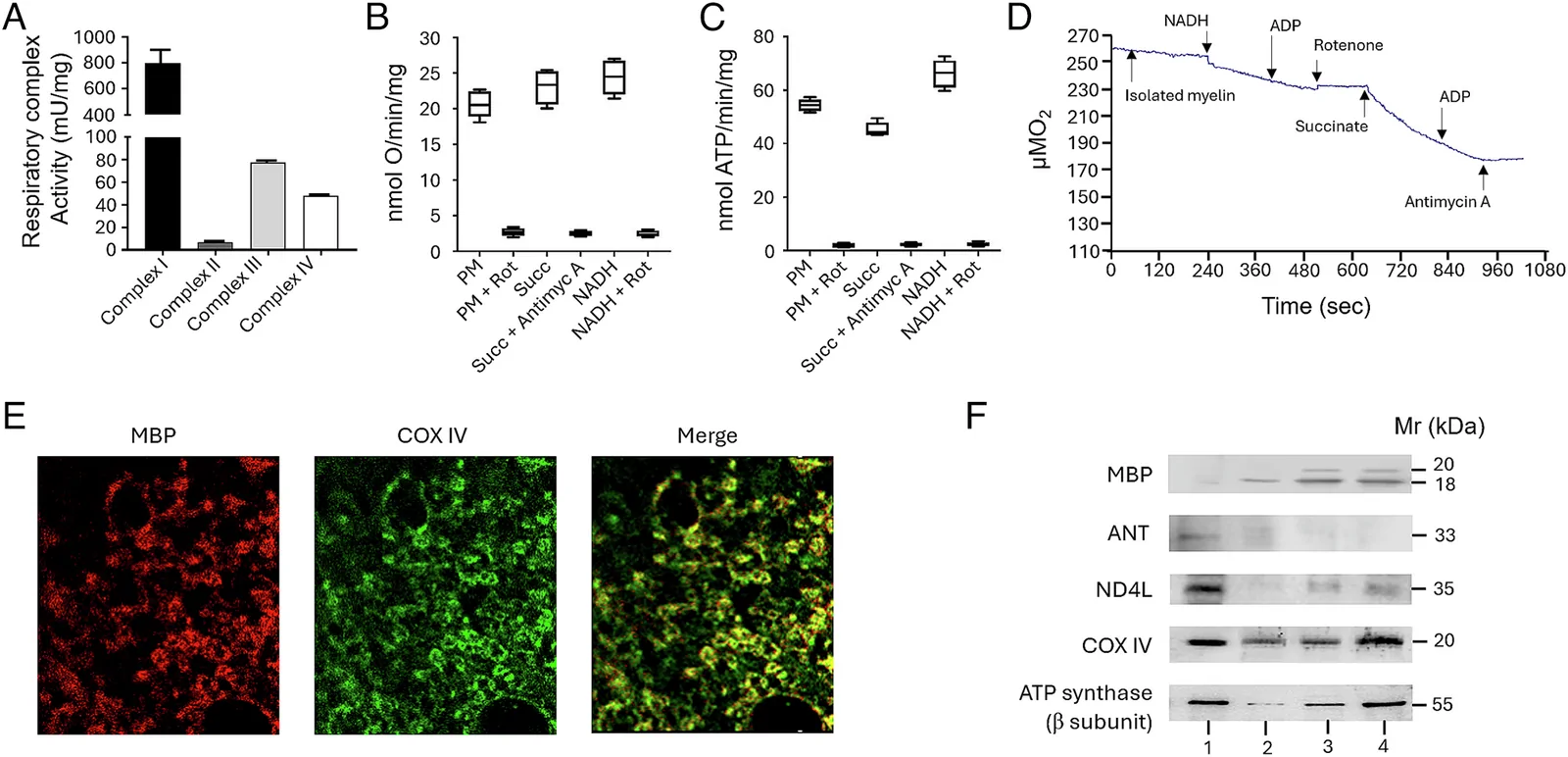
Functional expression of the five complexes of respiration in myelin. Panel *A*: Activity of Complexes I to IV of the Electron Transport Chain in human myelin fractions isolated from autoptic brain samples, according to the method of Norton and Poduslo (8); Panel *B*: Oxygen (O2) consumption rates, and Panel C: ATP synthesis in isolated myelin, induced by pyruvate + malate, succinate and the unconventional substrate NADH; rotenone and antimycin A, specific inhibitors of Complexes I and III, abolished respiration, and ATP production; Panel *D*: Representative trace of O_2_ consumption by isolated myelin, induced by NADH and succinate, plus 0.1 mM ADP, blocked by specific inhibitors, data in Panels *A*–*D* are mean ± SD from four experiments; *Panel E*: Expression of Myelin Basic Protein (MBP), a marker of myelin sheath, and subunit IV of Cytochrome *c* oxidase (COX IV) in human brain slices, stained by indirect fluorescence (secondary Ab conjugated to Alexa-488, for COX IV and to Cy3 for MBP), merged yellow fluorescence shows the colocalization of MBP and COX IV; Panel *F*: Western Blot of MBP, adenine nucleotide translocase (ANT), ND4L subunit of Complex I, and ATP synthase beta subunit in brain mitochondria-enriched fraction (lane 1), brain homogenate (lane 2), crude myelin (lane 3), isolated myelin (lane 4), Mw indicates the molecular weight markers. Each panel is representative of at least five experiments.

This hypothesis is consistent with earlier experimental observations suggesting that myelin is metabolically active (2). Isolated myelin membranes express functional cytochrome *c* oxidase (COX), sensitive to classical inhibitors and capable of consuming oxygen (3). As shown in Fig. 1, isolated myelin consumes oxygen and expresses COX activity, supporting the idea that the sheath itself may conduct aerobic metabolism. A progressive enrichment of COX with increasing myelin purification steps, in contrast to other mitochondrial proteins (Fig. 1), suggests selective localization of this respiratory Complex in myelin membranes, consistent with previous proteomic analyses (4). Locally produced ATP may be delivered to the axoplasm through connexon-mediated mechanisms (5). The concept of metabolically active myelin may also account for certain neuroimaging findings. Specifically, the more intense and prolonged negative initial dip in the blood oxygen level–dependent (BOLD) signal observed in white matter compared to gray matter could reflect active oxygen consumption within the myelin, rather than passive storage alone (6). Chronic demyelination would impair axonal ATP supply, providing a rationale for the observed axonal degeneration. Conversely, loss of myelin may reduce available oxygen buffering but not eliminate it. Notably, being rich in neutral lipids, which are excellent solvents for oxygen (7), the myelin sheath may function as an oxygen attractor and buffer, ultimately facilitating its continuous utilization by COX.

In conclusion, while we support the authors’ conclusion that increased myelination enhances the brain ability to meet heightened oxygen demands, we suggest that this effect may not rely solely on passive storage. The presence of COX in myelin, its capacity for oxygen consumption (Fig. 1), and the structural integration of multilamellar membranes suggest a metabolically active role for oxygen in myelin. Notably, the number of myelin wraps increases with axonal diameter (9), which may reflect a need for greater local metabolic support. We may say that myelin provides both physical and trophic support to neurons, serving as an electrical insulator and a dynamic oxygen buffer (2).
